# Letter from Dr. W. N. Cote

**Published:** 1864-11

**Authors:** 


### LETTERS FROM DR. W. N. COTE.

Foreign Correspondence of the Philadelphia Medical and Surgical Reporter.

PARIS, *June* 28, 1864.

*Albuminuria.*—In albuminuria the urine is of a very low specific gravity, never exceeding 1012, and sometimes falling as low as 1004, whilst the specific gravity of healthy urine is about 1015, and in diabetis melitus it often rises to 1040. This unnaturally low density of the urine in albuminuria shows that the other ingredients proper to healthy urine, such as urea and salts, are preternaturally diminished in quantity. Whilst the solid contents of healthy urine amounts to about seventy parts in a thousand, in albuminuria they are often reduced to twelve, and have been met with even lower than this. Various methods of treatment have been resorted to for diminising the quantity of albumen, an animal principle which is a great agent in nutrition and not an excrementitious product, and increasing the quantity of urea and salts which should be eliminated from the body. Mercury has been employed, but opinions vary as to the propriety of using it in the granular degeneration of the kidney, and although instances of recovery are recorded after severe salivation, the general impression is, that the mercurial influence is prejudicial rather than salutary. The tension of vessels is often relieved by cupping-glasses, and the secretions of the skin much promoted by the use of warm baths and diaphoretics, whilst purgation and diuretics have been employed for removing as far as possible the dropsical accumulation. A physician of this city, Dr. Namias, has lately had recourse to electricity in the treatment of this disease, and he has obtained very hopeful results from its application. This agent has, it appears, the property of increasing the quantity of urea in the urine. Whether it does so directly by acting on the urea itself, or indirectly by invigorating the whole economy has not yet been ascertained. I confine myself to stating the fact without commentary. It would be well could experiments be made with this powerful remedy in order to show its real value in the treatment of albuminuria.

*Variations of the Pulse in Lying-in Women.*—In lying-in women enjoying good health, a slackening of the pulse is usually met with. The frequency of this phénomenon necessarily varies according to the sanitary condition of the econemy. It does not depend upon any particular condition of some females, some of whom may have a quick pulse and others a slow pulse. According to Dr. Blot it is a general fact in relation with the depletion of the uterus. The degree of relaxation may vary considerably, it most usually oscillates between 44 to 60 pulsations. In one case the pulse fell as low as 35. The alimentary regimen seems to have no influence whatever on this phenomenon. It is more frequently met with in multiparous women than in primiparous ones, a fact which finds its explanation in the greater frequency of puerperal accidents in the latter class. The duration of the relaxation varies from a few hours to ten or twelve days. As a general rule the greater the relaxation the longer it lasts. The progress of this relaxation of the pulse is usually the same, it begins within twenty-four hours after the delivery, goes on increasing, remains stationary, and then dispears by degrees. It has often been seen to persist during the period so generally and yet improperly denominated as milk fever. This anomalous condition of the pulse seems to exert no very marked influence on the general state of the economy, although it may be considered as a very favorable symptom since it is met with only in healthy women. In an hospital its frequency indicates an excellent sanitary condition; its variety, on the contrary, must give reason to fear the near approach of some epidemic. As to its cause, it should not be sought in a kind of nervous exhaustion. The sphymographic researches made with the help of M. Marez, show in a manifest manner that it is in relation with an increase of arterial tension after delivery.

*Treatment of Epilepsy.*—Dr. Brown-Séquard, author of a number of fine works, most of which have been published in France, has shown the influence exercised by relax action on paralysis and convulsions. For many years past, he has endeavored to arrest epileptic fits, either by directly treating the part from whence proceeded the *aura*, or by interrupting the transmission of the convulsory starts by means of a ligature, which the patient promptly tightens the moment he feels the sensation marking the invasion of the fits. By employing this mode of treatment Dr. Brown-Séquard has been enabled to operate some remarkable cures.

*Rubeola.*—Rubeola has been long since known in Germany under the name of *Roetheln.* It was pointed out for the first time in France, in 1574, by Baillou. It has been observed under an epidemical form in Alsace, by Matthieu; in Haute Saxe, by Zeigler; at Groningue, by Geertsema. During an epidemic of scarlatina, which raged from 1838 to 1840, at Strasburg, Dr. Stoeber observed several cases of this disease, and gave it the name of *scarlatine rubeoleuse.* Drs. Barthoz and Rilliet, in their work on diseases of children have met both with scarlatina and measles in seven cases. In his *Traite de Pathologie*, Dr. Gintrac, of Bordeaux, devotes a special chapter to the study of this disease, and publishes five well detailed cases of it. Rubeola, according to our author, may be considered as having an existence of its own, and deserves a place apart in the pathology of the skin.

*Osteogenesis.*—Dr. Bruch, of Germany, has communicated to the Academy of Sciences the results of his researches on osteegenesis. The main conclusion our author derives from his study of this subject is as follows: "I consider it incontestable that the osseous tissue in all classes of vertebræ, is formed by *epigenesis*, that is, by successive layers which are of an osseous nature from their beginning, either at the external or internal part of the cartilages. The pretended ossification of the cartilage never produces bone, it is always cartilage impregnated with calcareous substances, the cellules of which always maintain the same form, and are never transformed into anastomatic radiary osseous corpuscles.

*Acetate of Potash in Urethral Blenorrhagic.*—Dr. Ambrosale, of Italy, is much against the use of acetate of potassa in the treatment of urethral blenorrhagia. Administered in heavy doses it cures acute and subacute blenorrhagic urethritis, but exercises no influence whatever on the other kinds of blenorrhagia. In order to obtain satisfactory results 100 grammes need be taken and even more. The injections made with a saturated solution of this salt modify the mucous coats and arrest the progress of the morbid secretion, but they act with slowness, so that they should not be preferred to those of sulphate of zinc, tannin, alum, etc. Even at high doses the acetate of potassa is well borne by the stomach, and only brings about an abundant secretion of urine. Owing to the slowness with which it acts, this salt cannot replace the balsamic substances nor the ordinary injections, nor again the means called abortive. It should be prescribed only to patients who cannot bear balsam or refuse to take injections. Whether employed internally or under the form of injection, acetate of potassa has no effect on chronic inflammations of the urethra. The action of this salt is purely topical and diuretic.

*Nitrate of Silver in Peraplegia*—Dr. Bouchut, physician to the Hospital St. Eugénie, recommends nitrate of silver against the essential paraplegia so often met with in children. He relates several cases in which this remedy has been used with the greatest success. His plan consists in administering at first one centigramme of nitrate of silver, divided into two pills, one in the morning and the other in the evening. The dose may be increased until a notable amelioration takes place. It is probable that the cases of paralysis adverted to by Dr. Bouchut would have been cured by themselves. Still it must be admitted that all means, except the nitrate of silver, have hitherto proved very unsatisfactory.

